# Updates on the Treatment of Pancreatic Diseases: Focus on Surgery, Electrochemotherapy and Rituximab

**DOI:** 10.3390/jcm11010239

**Published:** 2022-01-03

**Authors:** Romain Coriat, Anna Pellat

**Affiliations:** 1 Gastroenterology and Digestive Oncology Unit, AP-HP Centre, Cochin Hospital, 27 Rue du Faubourg Saint-Jacques, 75014 Paris, France; anna.pellat@aphp.Fr; 2 UFR de Médecine, Faculté de Santé, Université de Paris, 75006 Paris, France

The pancreas plays an important role in the human body with its two main endocrine and exocrine functions. Various types of disease can arise from the pancreas, but are mainly represented by pancreatitis and benign or malignant tumors.

Pancreatic cancer, mostly represented by pancreatic adenocarcinoma (PA), is the seventh cause of cancer death worldwide [1,2]. It has a particularly high toll in Western countries and its incidence has been rising in recent years, yet with a lack of knowledge of all risk factors [3,4]. Despite the development of various treatments (e.g., surgery, chemotherapy, and radiotherapy), the prognosis of PA remains very low, with a 5-year survival rate of approximately 10% [1]. Other pancreatic tumors include pancreatic neuroendocrine neoplasms (pNENs) and cystic neoplasms such as intraductal papillary mucinous neoplasms (IPMNs), serous neoplasms, mucinous neoplasms (MCNs) and solid pseudopapillary neoplasms. PNENs are considered malignant, with great clinical variability depending on their differentiation and grade, while IPMNs and MCNs can shift towards pancreatic malignancy. 

In the past three decades, there have been improvements in pancreatic surgical procedures. For patients with a localized pancreatic tumor involving the periampullary region, pancreatoduodenectomy (PD) is the recommended surgery, although there is still no standard regarding the surgical reconstruction method. In the present Special Issue, Bizzoca et al. found surgical morbidity and mortality rates of 26% and 6%, respectively, in a retrospective series of 100 patients treated with PD and reconstructed with wirsung-pancreato-gastro-anastomosis [5]. The overall rates of post-operative pancreatic fistula and delayed gastric emptying syndrome were low in this study [5]. They highlighted their results by comparing them with the available literature on wirsung-pancreato-gastro-anastomosis [5]. Furthermore, to reduce exocrine and endocrine complications, parenchymal-sparing pancreatic and duodenal resections have been considered for various benign or borderline malignant pancreatic tumors. Marchese et al. describe these different techniques in a mini review of this Special Issue offering tips and tricks for surgeons [6]. These surgical techniques, including enucleation, uncus resection, central pancreatectomy, ampullectomy and duodenectomy, present excellent long-term functional results [6]. 

As mentioned previously, the prognosis of PA remains very low and available treatment options are limited. New treatments such as targeted therapy and immunotherapy have been evaluated in selected populations of PA and are still being considered in ongoing trials [7,8]. Electrochemotherapy is the combination of electroporation (local application of an intensive electric field to increase the permeability of cell membranes) and chemotherapy. It has been considered for PA in the locally advanced setting to increase survival [9,10] but requires a laparotomy approach, which currently limits its applicability. In this Special Issue, Izzo et al. propose a protocol for a phase IIb prospective randomized trial that will compare chemotherapy with FOLFOXIRI versus electrochemotherapy with FOLFOXIRI in patients with locally advanced PA [11].

Pancreatitis is another frequent pancreatic disease. We can distinguish acute pancreatitis, usually caused by alcohol intake or gallstone disease [12], from chronic pancreatitis. Among chronic pancreatitis cases, two subtypes of chronic autoimmune pancreatitis (AIP) have been described. Type 1 AIP is part of the IgG4-related disease, which is a chronic fibro-inflammatory disorder that can affect many organs. The first-line treatment is usually steroids but with a risk of relapse and exposition to side effects over time. Rituximab, a B-cell-depleting antibody targeting CD20 surface antigen, is an established step-up therapy for type 1 AIP, but long-term data with this treatment are scarce. In this Special Issue, Backhus et al. describe a cohort of 46 patients with type 1 AIP, including 13 (28%) patients treated with rituximab [13]. Clinical activity significantly decreased in the short term after induction with rituximab [13]. Eight (61%) patients relapsed (median relapse-free survival of 16 months) but responded well to reinduction [13]. The safety profile of rituximab was acceptable [13].

Pancreatic diseases are frequent and usually require specific expertise with a multidisciplinary approach. With its incidence rising and its low prognosis, new therapeutics are urgently needed for PA. Other types of pancreatic tumors, as well as AIP, are rare conditions and require collaborative group effort to improve their management in the future. 
